# Repeating noninvasive risk stratification improves prediction of outcome in ICD patients

**DOI:** 10.1111/anec.12794

**Published:** 2020-08-17

**Authors:** Bert Vandenberk, Vincent Floré, Christian Röver, Mark A. Vos, Albert Dunnink, Dionyssios Leftheriotis, Tim Friede, Panagiota Flevari, Markus Zabel, Rik Willems

**Affiliations:** ^1^ Department of Cardiovascular Sciences University of Leuven Leuven Belgium; ^2^ Department of Cardiology University Hospitals Leuven Leuven Belgium; ^3^ Department of Medical Statistics University Medical Center Göttingen Gottingen The Netherlands; ^4^ Department of Physiology University Medical Center Utrecht, UMCU Utrecht The Netherlands; ^5^ Department of Cardiology Attikon University Hospital, AUH Athens Greece; ^6^ Department of Cardiology Heart Centre University Medical Center Göttingen Gottingen The Netherlands

**Keywords:** autonomic function, heart rate turbulence, left ventricular ejection fraction, microvolt T‐wave alternans, noninvasive risk stratification, sudden cardiac death

## Abstract

**Background:**

Noninvasive risk stratification aims to detect abnormalities in the pathophysiological mechanisms underlying ventricular arrhythmias. We studied the predictive value of repeating risk stratification in patients with an implantable cardioverter‐defibrillator (ICD).

**Methods:**

The EUTrigTreat clinical study was a prospective multicenter trial including ischemic and nonischemic cardiomyopathies and arrhythmogenic heart disease. Left ventricular ejection fraction ≤40% (LVEF), premature ventricular complexes >400/24 hr (PVC), non‐negative microvolt T‐wave alternans (MTWA), and abnormal heart rate turbulence (HRT) were considered high risk. Tests were repeated within 12 months after inclusion. Adjusted Cox regression analysis was performed for mortality and appropriate ICD shocks.

**Results:**

In total, 635 patients had analyzable baseline data with a median follow‐up of 4.4 years. Worsening of LVEF was associated with increased mortality (HR 3.59, 95% CI 1.17–11.04), as was consistent abnormal HRT (HR 8.34, 95%CI 1.06–65.54). HRT improvement was associated with improved survival when compared to consistent abnormal HRT (HR 0.10, 95%CI 0.01–0.82). For appropriate ICD shocks, a non‐negative MTWA test or high PVC count at any moment was associated with increased arrhythmic risk independent of the evolution of test results (worsening: HR 3.76 (95%CI 1.43–9.88) and HR 2.50 (95%CI 1.15–5.46); improvement: HR 2.80 (95%CI 1.03–7.61) and HR 2.45 (95%CI 1.07–5.62); consistent: HR 2.47 (95%CI 0.95–6.45) and HR 2.40 (95%CI 1.33–4.33), respectively). LVEF improvement was associated with a lower arrhythmic risk (HR 0.34, 95%CI 0.12–0.94).

**Conclusions:**

Repeating LVEF and HRT improved the prediction of mortality, whereas stratification of ventricular arrhythmias may be improved by repeating LVEF measurements, MTWA and ECG Holter monitoring.

## 1 INTRODUCTION

Implantable cardioverter‐defibrillators (ICDs) have been shown to improve survival in patients at risk of sudden cardiac death (Bardy et al., 2005; Moss et al., 2002). The majority of sudden cardiac deaths (SCD) occur in patients with normal or moderately impaired left ventricular ejection fraction (LVEF), whereas current primary prevention prophylactic ICD indications are mainly based on a severely impaired left ventricular function (LVEF ≤ 35%) (Huikuri, Castellanos, & Myerburg, 2001; Priori et al., 2015). SCD and ventricular arrhythmias result from a complex interplay of a myocardial substrate, the autonomic nervous system and myocardial vulnerability and triggers. There is an ongoing search for noninvasive risk stratification to guide the decision for ICD implantation, such as heart rate turbulence (HRT), microvolt T‐wave alternans (MTWA), and quantification of fibrosis on cardiac MRI (Bauer et al., 2008; Costantini et al., 2009; Huikuri et al., 2001). Recently, there has been increasing evidence on the association of the evolution in LVEF and the outcome of ICD patients (Schliamser et al., 2013; Zhang et al., 2015). Worsening of LVEF was associated with increased mortality and higher rate of appropriate ICD shocks, whereas LVEF improvement was associated with decreased mortality but the risk of appropriate ICD shocks remained present. The predictive value of the evolution of markers such as HRT and MTWA for outcome has not been studied extensively.

We report the findings of a subanalysis of the EUTrigTreat Clinical Study, a prospective multicenter observational study aiming to improve risk stratification of SCD in a broad population. The EUTrigTreat Clinical Study included repeating noninvasive testing and assessed the changes of noninvasive risk stratification tests and their prognostic value over time (Seegers et al., 2012).

## 2 METHODS

### 2.1 Study population

This analysis is part of the EUTrigTreat clinical study, which recruited 672 patients from January 2010 through April 2014 in 4 European centers (University Medical Center Utrecht – Vos M.A.; Attikon University Hospital – Flevari P.; University Hospitals Leuven – Willems R., Vandenberk B. and University Medical Center Göttingen – Zabel M., Friede T., Röver C.). All local ethics committees approved the study protocol. The study design, protocol, and main outcome have been published previously (Bergau et al., 2018b; Seegers et al., 2012). Briefly, all patients with a primary or secondary prophylactic indication for an ICD, who were at least 18 years of age were eligible for recruitment, unstable cardiac disease was excluded. Patients could be included either at first implant or during follow‐up. Baseline assessment included clinical characteristics, medical history, co‐morbidities, and drug treatment; laboratory samples, including renal function, high‐sensitive C‐reactive protein (hs‐CRP), and N‐terminal pro‐B‐type natriuretic protein (NT‐proBNP); echocardiography for LVEF; EP study and noninvasive ECG‐based risk stratification with microvolt T‐wave alternans testing and 24‐hr ECG Holter monitoring. It was recommended, but not mandatory, to repeat the noninvasive risk stratification between 6 months and 1 year after inclusion.

### 2.2 Microvolt T‐wave alternans testing

MTWA exercise testing was performed using the Cambridge Heart system (Cambridge Heart) if the patient was in sinus rhythm. The exercise intensity was gradually increased to reach a target heart rate of 110–120 beats per minute. If the patient was unable to exercise, heart rate could be increased gradually by a stepwise atrial pacing protocol in case of a dual‐chamber ICD or cardiac resynchronization therapy (Seegers et al., 2012). In patients receiving cardiac resynchronization therapy with underlying atrioventricular block, biventricular‐paced TWA was performed (Ehrlich et al., 2008). MTWA tests were graded in consensus by two blinded investigators from the enrolling and core centers according to the rules developed by Bloomfield, Hohnloser, and Cohen (2002). In case of disagreement, the finding was openly discussed, with the enrolling center in charge of the final grade. For further analysis of MTWA results, positive and indeterminate results were grouped as non‐negative.

### 2.3 24‐hr ECG Holter monitoring

A 24‐hr ECG Holter monitoring was performed using standard clinical recording devices (Delmar Reynolds Pathfinder, Spacelabs Healthcare; Ela Medical; GE Mars, GE Healthcare). Data were analyzed for the number of premature ventricular complexes (PVC) and nonsustained ventricular tachycardias normalized to 24 hr. The dichotomization of PVCs was performed based on the median number of PVCs on the 24‐hr ECG Holter recordings. HRT was calculated using dedicated software (Librasch Calc, V1.02, Schneider R and Schmidt G, TU Munich, Germany) (Schmidt et al., 1999). Turbulence onset <0% and turbulence slope >2.5 ms/RR‐interval were defined as normal (Bauer et al., 2008). A normal HRT test result was defined as both a normal turbulence onset and a normal turbulence slope, all other test results were considered abnormal. Exclusion criteria for HRT analysis were absence of sinus rhythm and atrial pacing in >15% of recorded RR intervals. A summary of 24‐hr ECG Holter registrations is available in Table S1 (Bergau et al., 2018a, 2018b).

### 2.4 Endpoints

From the predefined study endpoints, all‐cause mortality and first appropriate ICD shock were selected for analysis (Seegers et al., 2012). Patients were seen in the ambulatory ICD clinic every 3–6 months, or urgently in case of complaints. If an ICD shock occurred, the ECG data were stored and forwarded to the endpoint committee for classification. Due to the wide range of ICD indications and clinical characteristics, mandatory programming was not considered feasible by the investigators and programming was left to the treating physician. In the event of a patient's death, all available written information was retrieved.

### 2.5 Statistical analysis

All continuous variables are reported as median with the 25th and 75th percentiles, categorical and dichotomized variables as absolute counts and proportions (%). Demographics between groups were compared with chi‐squared test for categorical variables and Kruskal–Wallis test for continuous variables. The variables of interest were dichotomized to binary variables in which respectively a LVEF ≤ 40%, non‐negative MTWA, PVCs > 400, and abnormal HRT on a 24‐hr ECG Holter monitoring were considered high‐risk factors. Cox proportional hazards regression analysis was used to estimate the risk difference between patients with or without outcome events. For analysis of shocks, death was considered a censoring event (Therneau & Grambsch, 2000) using competing risk adjustments proposed by Fine and Gray (Fine & Gray, 1999). Adjusted analysis was adjusted for known independent predictors of shocks (LVEF and secondary prevention indication) and mortality (LVEF, atrial fibrillation, NT‐proBNP, NYHA class, and eGFR). The analysis was repeated comparing the status of the patients showing improvement (from high to low risk) or worsening (from low to high risk) of test results with consistent, both low and high risk, test results. Additionally, consistent low risk was compared with consistent high risk as positive control. Kaplan–Meier curves were compared using the log‐rank test. Computations were performed using the R environment for statistical computing and graphics (http://www.r‐project.org). All *p*‐values were two‐tailed, and α‐level of 0.05 was considered significant.

## 3 RESULTS

### 3.1 Demographics

A total of 635 patients had analyzable baseline results. The median age was 64.0 years (54.9–72.2), and 19% were female. Of these, 60 (9.4%) patients received their first implant at enrollment, the remaining had an ICD implanted 2.9 years (0.8–5.8) before. The overall follow‐up was 4.4 (3.2–5.3) years. Patient baseline characteristics and parameters are shown in detail in Tables 1 and 2. In total, 96 patients received at least 1 appropriate ICD shock during follow‐up which corresponds to an annual shock rate of 3.9%/year. There were 108 deaths resulting in an annual mortality of 4.0%/year.

**TABLE 1:** Baseline characteristics

	All patients
*n*	635
Age (years)	64.0 (54.9–72.2)
Primary prevention	400 (63%)
Female gender	122 (19%)
BMI (kg/m^2^)	27.1 (24.4–31.0)
LVEF (%)	40.0 (30.0–51.0)
CRT‐D	133 (21%)
Cardiac disease	
ICM	269 (42%)
DCM	216 (34%)
Other	150 (24%)
Idiopathic VT/VF	52 (8%)
HCM/HOCM	39 (6%)
Brugada syndrome	11 (2%)
LQTS	8 (1%)
ARVC	8 (1%)
Congenital	7 (1%)
Sarcoidosis	6 (1%)
Valvular cardiomyopathy	4 (1%)
CPVT	2 (1%)
Noncompaction CMP	2 (1%)
Other	11 (2%)
NYHA	
NYHA I	188 (30%)
NYHA II	265 (42%)
NYHA III	182 (28%)
Atrial fibrillation	
None	405 (65%)
Paroxysmal	137 (22%)
Permanent	80 (13%)
NT‐proBNP (ng/L)	646.0 (214.5–1,566.8)
Hs‐CRP (mg/L)	2.0 (1.0–5.0)
eGFR (mL/min)	71.3 (56.1–88.5)
Follow‐up (year)	4.4 (3.2–5.3)
Appropriate shock	96 (15%)
Death	108 (17%)
Repeated measures available	
LVEF	359 (57%)
MTWA	268 (42%)
PVC/24 hr	393 (62%)
HRT	200 (32%)

Abbreviations: ARVC, arrhythmogenic right ventricular cardiomyopathy; BMI, body mass index; CPVT, catecholaminergic polymorphic ventricular tachycardia; CRT‐D, cardiac resynchronization therapy—defibrillator; DCM, dilated cardiomyopathy; eGFR, estimated glomerular filtration rate; HCM/HOCM, hypertrophic (obstructive) cardiomyopathy; HRT, heart rate turbulence; hs‐CRP, high‐sensitive C‐reactive protein; ICM, ischemic cardiomyopathy; LQTS, long QT‐syndrome; LVEF, left ventricular ejection fraction; MTWA, microvolt T‐wave alternans; NT‐proBNP, brain natriuretic peptide; NYHA, New York Heart Association; PVC, premature ventricular complexes; VT/VF, ventricular tachycardia/ ventricular fibrillation.

**TABLE 2:** Baseline characteristics by cardiac disease

	ICM (*N* = 269)	DCM (*N* = 216)	Other (*N* = 148)	*p*‐value
Age (years)	68.0 (60.8–73.8)	63.6 (55.1–72.7)	54.3 (43.6–65.1)	<.001
Primary prevention	158 (59%)	177 (82%)	66 (45%)	<.001
Female gender	30 (11%)	39 (18%)	52 (35%)	<.001
BMI (kg/m^2^)	27.5 (24.6–30.9)	27.5 (24.9–31.5)	26.0 (23.9–30.0)	.009
LVEF (%)	35.0 (28.0–45.0)	35.0 (27.0–45.0)	55.0 (46.5–60.0)	<.001
CRT‐D	55 (20%)	74 (34%)	4 (3%)	<.001
NYHA				
NYHA I	55 (21%)	54 (25%)	79 (53%)	<.001
NYHA II	122 (45%)	97 (45%)	44 (30%)	
NYHA III	92 (34%)	65 (30%)	25 (17%)	
Atrial fibrillation				
None	178 (67%)	123 (59%)	104 (72%)	<.001
Paroxysmal	56 (21%)	44 (21%)	36 (25%)	
Permanent	32 (12%)	43 (20%)	5 (3%)	
NT‐proBNP (ng/L)	759.0 (279.0–1,777.5)	731.0 (280.0–1,879.3)	286.0 (99.5–780.0)	<.001
hsCRP (mg/L)	2.1 (1.0–5.0)	2.2 (1.0–5.0)	1.7 (1.0–3.45)	.023
eGFR (mL/min)	67.9 (52.4–82.3)	69.6 (55.4–88.8)	80.3 (64.4–95.6)	<.001
Appropriate shock	45 (17%)	28 (13%)	23 (16%)	.520
Death	52 (19%)	51 (24%)	5 (3%)	<.001

Abbreviations: BMI, body mass index; CRT‐D, cardiac resynchronization therapy—defibrillator; DCM, dilated cardiomyopathy; eGFR, estimated glomerular filtration rate; hs‐CRP, high‐sensitive C‐reactive protein; ICM, ischemic cardiomyopathy; LVEF, left ventricular ejection fraction; NT‐proBNP, brain natriuretic peptide; NYHA, New York Heart Association.

### 3.2 LVEF

In 359 patients (57%), at least 2 measurements of LVEF were available for further analysis with a median interval of 7.0 months (5.9–11.9) between measurements (Table S2). LVEF changed only in 53 (15%) patients. It deteriorated in 13 (4%) and improved in 40 (11%). Patients in whom LVEF deteriorated or that had a repeated LVEF ≤ 40% had higher NT‐proBNP levels and worse renal function at baseline compared to patients with improvement of LVEF.

**FIGURE 1:**
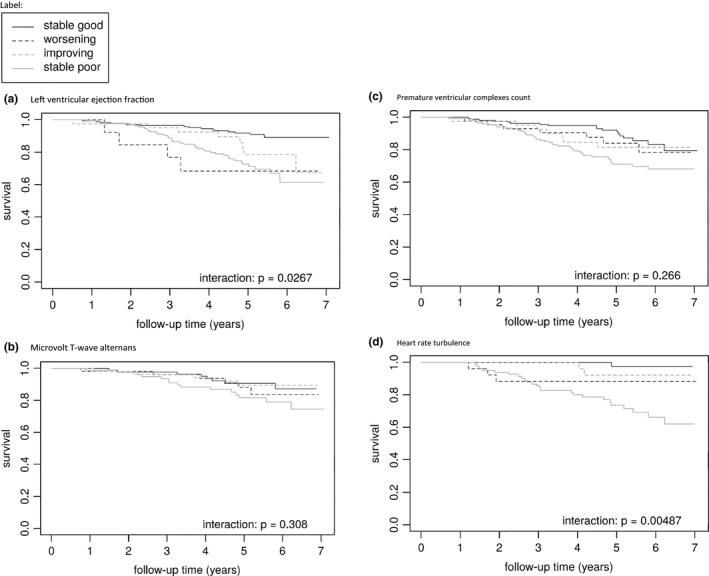
Kaplan–Meier analysis for mortality

Kaplan–Meier analysis showed a significant interaction for the evolution of LVEF on survival (*p* = .005) as shown in Figure 1a. Unadjusted and adjusted Cox regression for mortality are presented in Tables 3 and 4. A baseline reduced LVEF was an independent predictor of mortality, as were worsening of LVEF and consistent low LVEF.

**TABLE 3:** Unadjusted Cox regression modeling for mortality and appropriate ICD shock. (A) Mortality. (B) Appropriate ICD shocks

(A)						
	Baseline	Worsening		Improvement		Unchanged
	high risk versus low risk	versus stable low risk	versus stable high risk	versus stable low risk	versus stable high risk	high risk versus low risk
LVEF	2.89 (1.71, 4.91)	4.81 (1.60, 14.44)	1.26 (0.45, 3.53)	2.38 (1.02, 5.58)	0.63 (0.29, 1.34)	3.82 (2.12, 6.87)
MTWA	1.43 (0.72, 2.84)	1.28 (0.44, 3.69)	0.64 (0.25, 1.65)	0.97 (0.32, 2.98)	0.49 (0.18, 1.34)	2.00 (0.85, 4.72)
PVC	2.12 (1.31, 3.44)	1.47 (0.62, 3.53)	0.57 (0.26, 1.28)	1.51 (0.63, 3.61)	0.59 (0.26, 1.31)	2.57 (1.48, 4.46)
HRT	5.35 (1.87, 15.33)	7.52 (0.78, 72.34)	0.38 (0.12, 1.27)	4.14 (0.38, 45.66)	0.21 (0.05, 0.89)	19.69 (2.66, 145.60)

(B)						
	Baseline	Worsening		Improvement		Unchanged
	high risk versus low risk	versus stable low risk	versus stable high risk	versus stable low risk	versus stable high risk	high risk versus low risk
LVEF	1.88 (1.14, 3.10)	2.21 (0.63, 7.75)	0.91 (0.26, 3.12)	0.82 (0.28, 2.36)	0.34 (0.12, 0.94)	2.43 (1.44, 4.12)
MTWA	1.34 (0.73, 2.48)	3.69 (1.40, 9.68)	1.49 (0.68, 3.27)	2.80 (1.03, 7.61)	1.13 (0.50, 2.59)	2.47 (0.95, 6.45)
PVC	1.86 (1.15, 3.03)	2.52 (1.15, 5.49)	1.04 (0.52, 2.11)	2.46 (1.07, 5.65)	1.02 (0.48, 2.17)	2.41 (1.34, 4.36)
HRT	1.03 (0.54, 2.00)	0.82 (0.27, 2.51)	0.81 (0.27, 2.39)	0.88 (0.31, 2.51)	0.86 (0.31, 2.40)	1.01 (0.47, 2.19)

Abbreviations: HRT, heart rate turbulence; ICD, implantable cardioverter‐defibrillator; LVEF, left ventricular ejection fraction; MTWA, microvolt T‐wave alternans; PVC, premature ventricular complexes.

**TABLE 4:** Adjusted Cox regression modelling for mortality and appropriate ICD shock. (A) Mortality. (B) Appropriate ICD shocks

(A)						
	Baseline	Worsening		Improvement		Unchanged
	high risk versus low risk	versus stable low risk	versus stable high risk	versus stable low risk	versus stable high risk	high risk versus low risk
LVEF	1.79 (1.03, 3.13)	3.59 (1.17, 11.04)	1.69 (0.58, 4.95)	1.98 (0.84, 4.65)	0.93 (0.42, 2.04)	2.13 (1.14, 3.97)
MTWA	0.60 (0.27, 1.34)	0.77 (0.24, 2.51)	1.36 (0.50, 3.66)	0.39 (0.09, 1.64)	0.68 (0.18, 2.50)	0.57 (0.19, 1.70)
PVC	1.28 (0.74, 2.19)	1.04 (0.38, 2.86)	0.74 (0.28, 1.91)	0.90 (0.35, 2.29)	0.64 (0.26, 1.53)	1.41 (0.76, 2.61)
HRT	2.45 (0.81, 7.46)	4.11 (0.40, 42.07)	0.49 (0.14, 1.78)	0.86 (0.05, 14.66)	0.10 (0.01, 0.82)	8.34 (1.06, 65.54)

(B)						
	Baseline	Worsening		Improvement		Unchanged
	high risk versus low risk	versus stable low risk	versus stable high risk	versus stable low risk	versus stable high risk	high risk versus low risk
LVEF	2.26 (1.33, 3.84)	2.21 (0.63, 7.75)	1.00 (0.29, 3.45)	0.82 (0.28, 2.36)	0.34 (0.12, 0.94)	2.43 (1.44, 4.12)
MTWA	1.12 (0.59, 2.12)	3.76 (1.43, 9.88)	1.52 (0.69, 3.34)	2.80 (1.03, 7.61)	1.13 (0.50, 2.59)	2.47 (0.95, 6.45)
PVC	1.61 (0.98, 2.65)	2.50 (1.15, 5.46)	1.04 (0.52, 2.11)	2.45 (1.07, 5.62)	1.02 (0.48, 2.17)	2.40 (1.33, 4.33)
HRT	0.91 (0.45, 1.83)	0.82 (0.27, 2.51)	0.80 (0.27, 2.36)	0.88 (0.31, 2.51)	0.85 (0.31, 2.37)	1.03 (0.48, 2.22)

Note

Mortality: adjusted for LVEF, AF, NT‐proBNP, NYHA, eGFR. Appropriate ICD shocks: adjusted for LVEF, secondary prevention.

Abbreviations: AF, atrial fibrillation; eGFR, estimated glomerular filtration rate; HRT, heart rate turbulence; ICD, implantable cardioverter‐defibrillator; LVEF, left ventricular ejection fraction; MTWA, microvolt T‐wave alternans; NT‐proBNP, brain natriuretic peptide; NYHA, New York Heart Association; PVC, premature ventricular complexes.

**FIGURE 2:**
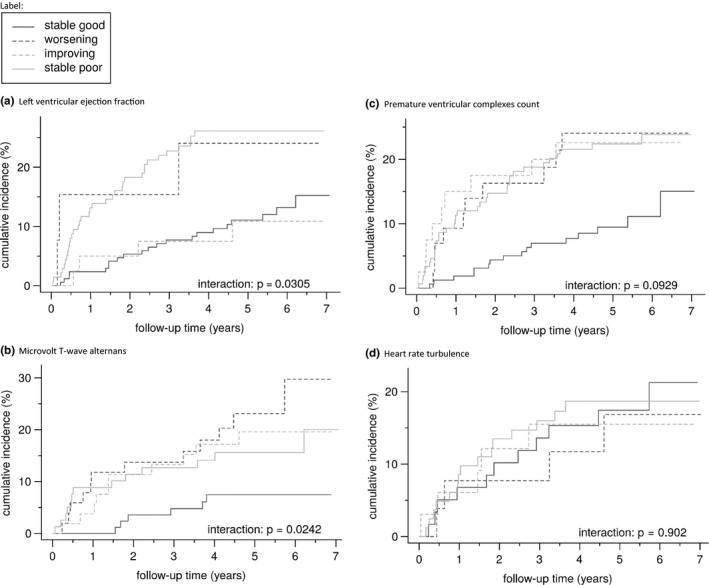
Kaplan–Meier analysis for appropriate ICD shocks

The Kaplan–Meier analysis for appropriate ICD shocks showed a significant difference between subgroups for shock‐free survival (*p* = .031, Figure 2a). In unadjusted analysis, baseline low LVEF was associated with appropriate ICD shocks, whereas LVEF improvement was associated with a reduced risk compared to a consistently low LVEF. After adjusted analysis baseline low LVEF remained an independent predictor for appropriate ICD shocks as did consistently low versus consistently preserved LVEF. LVEF improvement remained independently associated with a decreased risk for appropriate ICD shocks.

### 3.3 MTWA

In 268 patients (42%), at least 2 measurements of MTWA were available for further analysis (Table S3). The interval between tests was 6.2 months (5.5–7.2). Serial consistency of MTWA was observed in 62%. A total of 51 (19%) patients had worsening and 53 (20%) improvement of the test result. Patients with a positive MTWA test at baseline had a significantly lower LVEF and had a higher NYHA class. Patients with at least one positive MTWA test were significantly older and had higher NT‐proBNP levels at baseline.

Unadjusted analysis for mortality showed only a trend toward significantly increased risk comparing consistent non‐negative with consistent negative MTWA. This trend was no longer found in adjusted analysis. Kaplan–Meier analysis showed no significant interaction between survival and test results (*p* = .308).

For appropriate ICD shocks, Kaplan–Meier analysis showed a significant interaction with higher shock rates in patients with ≥1 non‐negative test (*p* = .024) independent of the evolution in test results. A single baseline MTWA test was no independent predictor of ICD shocks; however, consistent negative testing was associated with a decreased risk of appropriate ICD shocks compared with changing MTWA test results, that is, at least one non‐negative test. Consistent non‐negative testing showed a trend toward a higher risk of appropriate ICD shocks in adjusted analysis.

### 3.4 PVC

In 393 patients (62%), at least two 24‐hr ECG Holter recordings were available for analysis of PVC count (Table S4). The interval between tests was 6.7 months (5.8–10.7). In 79% of patients, the PVC count was comparable on the 2 sequential 24‐hr Holter ECG recordings. Of the remaining patients, 43 (11%) had more PVCs at the second recording, while 40 (10%) had less. Patients with at least one test with >400 PVCs/24 hr were significantly older, had higher NYHA class and higher baseline NT‐proBNP levels.

Unadjusted Cox regression for mortality showed an association of a high PVC count with mortality, both at baseline and when analyzing a consistent high PVC count. Improvement or worsening of PVCs yielded no additional predictive value (*p* = .266, Figure 1c). In adjusted analysis, there was no significant association.

For appropriate ICD shocks, unadjusted analysis identified patients with ≥1 24‐hr Holter ECG recording with high PVC count at any time at increased risk, both at baseline and at follow‐up. In adjusted analysis, patients with ≥1 high PVC count at any time out of 2 tests were at increased risk of appropriate ICD shocks. The result of a single test at baseline showed a trend toward significance. In Kaplan–Meier analysis, the interaction between PVC count and appropriate ICD shock showed a nonsignificant trend (*p* = .093, Figure 2c).

### 3.5 HRT

Sequential HRT analysis was available in 200 patients (32%) (Table S5). The interval between tests was 6.3 months (5.7–9.4). HRT results were consistent in 71% of patients and 26 (13%) had worsening of HRT and in 33 (16%) HRT improved. Patients with two normal HRT measurements had a higher baseline LVEF, were significantly older, and had a lower NT‐proBNP and a better renal function.

Kaplan–Meier analysis showed a significant interaction between HRT results and survival (*p* = .005, Figure 1d). Patients with improvement of HRT test results had a significant lower risk of dying when compared to patients with a consistent abnormal HRT result. Also, a consistent abnormal HRT test result at follow‐up was associated with a significant higher mortality when compared to consistent normal testing, a risk not present in adjusted analysis for baseline HRT results. For appropriate ICD shocks, there was no additional value in retesting HRT (*p* = .902, Figure 2d).

## 4 DISCUSSION

This prospective study aimed at investigating the evolution of noninvasive risk stratification tests and the additional clinical value of repeating risk stratification. For predicting mortality, adjusted analysis showed that repeating LVEF measurements and HRT testing could add prognostic value to standard risk stratification at baseline. Worsening of LVEF despite therapy was associated with a significant increase in mortality risk. A consistent abnormal HRT was an independent predictor of mortality, whereas patients with improved HRT test results had a lower mortality when compared with patients with consistent abnormal HRT testing.

For predicting appropriate shocks, improvement in LVEF was associated with a lower risk of appropriate shocks. Despite the fact that a high PVC count or non‐negative MTWA at baseline had no significant predictive value for appropriate ICD shocks in adjusted analysis, after repeating the test a high PVC count or a non‐negative MTWA test at any of the 2 measurements was associated with an increased risk of appropriate ICD shocks in our population, stressing the importance of repeating noninvasive risk‐evaluation.

### 4.1 LVEF

The proportion of patients with changed LVEF is lower than previously reported. However, our study results should be interpreted as long‐term evolution in LVEF as most of the patients in our study were not included at first device implant and a significant proportion of patients had a normal LVEF. Assuming that all patients in our serial LVEF analysis with a primary prevention indication in ICM or DCM had an LVEF ≤ 35% at first implant as demanded by guidelines (Priori et al., 2015), the fact that 27% of these patients had a LVEF > 40% at the baseline measurement, implies that a significant proportion had recovery of left ventricular dysfunction after initial implantation. This would correspond to previous reported data with rates of 14.3% up to 25.5% and 27.5% of patients, which no longer met primary prevention indications at generator change (Kini et al., 2014; Naksuk et al., 2013; Vandenberk et al., 2017). Although improvement of LVEF was associated with a 3‐times lower risk of appropriate ICD shocks, the remaining risk after LVEF recovery was not zero and persisted as was also shown in recent studies (Naksuk et al., 2013; Vandenberk et al., 2017). Worsening of LVEF to ≤40% was associated with a 3.5‐times higher mortality. In a substudy of the DEFINITE trial on repeated LVEF measurements, a >5% improvement of LVEF was associated with a significant lower mortality risk (HR 0.22, 95% CI 0.06–0.82, *p* = .023) and a trend to lower arrhythmic risk (HR 0.47, 95% CI 0.22–1.02, *p* = .055) (Schliamser et al., 2013). In the PROSE‐ICD study, including both ICM and NICM patients, LVEF improvement with >5% was associated with a lower risk of mortality (HR 0.33, 95% CI 0.18–0.59) and appropriate shocks (HR 0.29, 95% CI 0.11–0.78) (Zhang et al., 2015). In patients with LVEF worsening with >5% the risk of mortality (HR 1.54, 95% CI 0.87–2.75) and appropriate shocks (HR 0.51, 95% CI 0.05–5.30) remained similar. Further, the degree of LVEF recovery after a first MI was associated with sudden cardiac death and overall mortality (Chew et al., 2018). Therefore, we can conclude that an improvement in LVEF might be associated with a better arrhythmic and total survival, while a worsening in LVEF might be associated with a worse total survival without clear increase in arrhythmic risk. Hence, the timing of follow‐up LVEF measurements and the effects of heart failure therapy are crucial to determine optimal timing of ICD implantations.

### 4.2 MTWA

MTWA showed a limited consistency of 61% in this large number of patients. Long‐term consistency in 22 ICD patients with ICM or nonischemic cardiomyopathy was 76.6% with an interval of 11.8 ± 3.3 months between tests (Wierzbowski et al., 2007). Our study is, to our knowledge, the largest study investigating the clinical value of repeating MTWA testing. For mortality, the trend in unadjusted analysis for two non‐negative MTWA tests was not confirmed in adjusted analysis. For appropriate ICD shocks, a single baseline test yielded no predictive value; however, ≥1 non‐negative result out of 2 MTWA tests was associated with an increased risk for ICD shocks. In the MASTER trial, including ischemic cardiomyopathy patients with a LVEF ≤ 30%, a non‐negative MTWA test was associated with increased mortality (HR 2.04, 95% CI 1.10–3.78), but not with ventricular arrhythmias (HR 1.26, 95% CI 0.76–2.09) (Chow et al., 2008). In the ALPHA study, including nonischemic cardiomyopathy patients with a LVEF ≤ 40%, a non‐negative MTWA test was associated with an increased risk of cardiac mortality or ventricular arrhythmia (HR 3.21, 95% CI 1.12–9.22) (Salerno‐Uriarte et al., 2007).

### 4.3 PVC count

The predictive value of a high PVC count on 24‐hr Holter ECG recordings was shown previously in the Cardiovascular Health Study, including 1,139 participants aged 65 years or older with normal LVEF (Dukes et al., 2015). Patients in the upper quartile of the PVC count were compared with the lowest quartile and showed a 31% increased risk for heart failure (HR 1.48, 95% CI 1.08–2.04, *p* = .02) and mortality (HR 1.31, 95% CI 1.06–1.63, *p* = .01). In MADIT II, the predictive value was studied based on 10 min ECG Holter recordings with a cutoff value of 3/10 min or 432/24 hr (Berkowitsch et al., 2004). In the conventional study arm, a high PVC count was associated with a 63% increased risk of mortality (HR 1.63, *p* = .07) and in the ICD arm with a 75% increased risk of appropriate ICD therapy (HR 1.75, *p* = .003). Our study showed that a high PVC count on one of both ECG Holter recordings was associated with a significant increased risk of ventricular arrhythmias; however, for mortality the results in unadjusted analysis were no longer present after adjusting for covariates.

### 4.4 HRT

In our study, the baseline and repeated measurement of HRT did not show any predictive value for appropriate shocks, but patients with 2 abnormal HRT tests showed worse survival and improvement of HRT test results was associated with improved survival. A recent systematic review showed a consistent significant predictive value for abnormal HRT in the prediction of cardiac mortality and ventricular arrhythmias in patients postmyocardial infarction or heart failure patients (Disertori, Mase, Rigoni, Nollo, & Ravelli, 2016). Both CARISMA and REFINE studied the evolution of HRT after myocardial infarction. These trials identified recovery of HRT as a marker of patient recovery associated with LVEF increase after myocardial infarction (Exner et al., 2007; Huikuri et al., 2010). A lack of recovery of TS was a powerful predictor of life‐threatening arrhythmic events with an 8.4 times increase (95% CI 1.1–64.2, *p* = .03) in CARISMA and 5.9 times (95% CI 1.3–25.6, *p* = .009) in REFINE. These results are not translatable to the current study as most patients in the EUTrigTreat study were tested long after the primary cardiac event or diagnosis for which an ICD was implanted. In nonischemic heart disease, the role of HRT seems limited and failed to predict arrhythmic events (Bauer et al., 2008). In structural normal heart disease and HCM, the evidence is too limited for interpretation.

### 4.5 Clinical implications

The goal of noninvasive risk stratification test is to identify patients at high risk of life‐threatening ventricular arrhythmias but at low risk of nonarrhythmic mortality. The current guidelines provide adequate recommendations and decisions about ICD implants should be made on a case‐by‐case basis. Based on our current results repeating 24‐hr Holter ECG monitoring, MTWA or echocardiography when faced with a difficult clinical decision to implant or replace a defibrillator in patients questioning the indication, with borderline indications or significant comorbidity seems to be of clinical value. In our opinion, if an improvement in LVEF is found before first implantation, the procedure should be delayed or canceled. If it is noticed before replacement however, we would currently still consider the patient for replacement despite their improved prognosis because other studies showed that the incidence of appropriate shocks, hence the arrhythmic risk, remains clinically important (Schliamser et al., 2013; Vandenberk et al., 2017; Zhang et al., 2015). When severe deterioration of LVEF is documented during follow‐up chances of dying a nonsudden death increases and, certainly in patients with other co‐morbidities, this should be discussed openly with the patients before replacement. In patients with doubts about the indication or a borderline LVEF, any high PVC count on repeated 24‐hr Holter ECG recordings or non‐negative MTWA test during further follow‐up might be useful to identify the highest risk patients.

We believe our results show promise for developing a practical algorithm of repeated risk stratification to guide the decision on when and whether or not a patient should receive a first ICD implantation or ICD replacement. Combining noninvasive risk stratification tests according to their physiological background and their predictive value, based on optimal sensitivity and/or specificity can improve the risk prediction of SCD and mortality (Exner et al., 2007; Vandenberk et al., 2019). Of course, this should be tested in a prospective trial comparing it with current guidelines and determine whether it could expand ICD indications beyond LVEF or minimize implants in patients with a high nonarrhythmic mortality risk and low arrhythmic risk.

### 4.6 Limitations

The EUTrigTreat Clinical Study was a prospective multicenter observational study powered to risk stratify a large cohort of diverse ICD recipients for all‐cause mortality and appropriate ICD shocks using noninvasive risk stratification tools (Seegers et al., 2012). Part of the study objective was studying noninvasive test dynamicity and their predictive value over time; however, other dynamic parameters such as renal function or prescription of anti‐arrhythmic drugs could not be included in the current analysis. Although the largest numbers of repeated tests in literature were compiled in this study, the study could not be powered to identify significant predictive differences for each test discussed above. Despite the fact that investigators were encouraged to repeat testing every 6 months, not all tests were available in all patients. Furthermore, the underlying cardiac cause was heterogeneous and both primary and secondary prevention ICD patients were included. However, subgroup analysis was not performed due to the risk of overinterpretation. Last, part of the patients were included at generator change and therefore certain selection bias might have been introduced since patients who died earlier were not included.

## 5 CONCLUSIONS

The dynamic risk of arrhythmias and mortality may be better assessed by repeating noninvasive risk stratification tests. There is a potential value of repeating LVEF measurements and HRT analysis in the prediction of mortality, whereas noninvasive risk stratification of ventricular arrhythmias may be improved by repeating LVEF measurements, MTWA and ECG Holter monitoring. Algorithms to guide decisions on timing of ICD implantation or extension of ICD therapy incorporating these tests warrant further investigation.

## CONFLICTS OF INTEREST

None.

## AUTHOR CONTRIBUTIONS

All authors reviewed and approved the manuscript.

*Directed this study:* M. Zabel, R. Willems, M. Vos, P. Flevari, T. Friede

*Collected patient data:* B. Vandenberk, V. Floré, A. Dunnink, D. Leftheriotis

*Performed statistical analysis:* C. Röver, T. Friede

*Wrote the main manuscript:* B. Vandenberk, R. Willems

*Gave suggestions on the manuscript:* all authors

## 6 ETHICS

Ethical approval was obtained from the local Ethical Review Board at each of the participating centers: University Medical Center, Göttingen, University Medical Center Utrecht, University Hospital Attikon, University Hospitals Leuven. The study was carried out in accordance with the Helsinki Declaration, as revised 2013.

## Supporting information

Supplementary Material
