# Supplementary material for: Repeating noninvasive risk stratification improves prediction of outcome in ICD patients
Source: Ann Noninvasive Electrocardiol. 2020 Aug 17;25(6):e12794. doi: 10.1111/anec.12794 (PMC7679829; doi:10.1111/anec.12794)
Supplement: Supplementary file 1 — Supplementary Material [file ANEC-25-e12794-s001.docx]

**Table S1.** Summary of 24-hour ECG Holter registration results.

|  | baseline | followup |
| --- | --- | --- |
| Median ECG Holter duration (Q1 – Q3) | 24.0 (23.0 – 24.0) | 24.0 (23.0 – 24.0) |
| Median number of PVCs (Q1 – Q3) | 385 (82 – 1791) | 395 (66 – 2000) |
| Mean number of ns-VT | 2.34 | 3.61 |
| Median number of ns-VT (Q1 – Q3) | 0.0 (0.0 – 0.0) | 0.0 (0.0 – 0.0) |
| Number (%) of patients with ≥1 ns-VT | 144 (22.8%) | 71 (18.3%) |
| Median HRT onset (Q1 – Q3) | -0.025 (-1.12 – 0.48) | -0.46 (-1.73 – 0.42) |
| Median HRT slope (Q1 – Q3) | 3.67 (2.05 – 7.50) | 3.550 (1.73 – 6.57) |
| Median maximal HR exercise MTWA (Q1 – Q3) | 110 (100 – 114) | 109 (98 – 114) |
| Median maximal negative HR exercise MTWA (Q1 – Q3) | 105 (92 – 111) | 105 (91 – 111) |

Data are presented as median with quartiles Q1 and D3 or mean as mentioned.

Abbreviations:

ECG: electrocardiogram; HRT: heart rate turbulence; MTWA: microvolt T-wave alternans; ns-VT: nonsustained ventricular tachycardia; PVC: premature ventricular complex

**Table S2.** Baseline characteristics by LVEF

|  | **Stable > 40%** | **Deterioration** | **Improvement** | **Stable ≤ 40%** | **p-value** |
| --- | --- | --- | --- | --- | --- |
| n (%) | 169 (47%) | 13 (4%) | 40 (11%) | 137 (38%) |  |
| Age (y) | 62.2 (52.3 – 70.1) | 60.8 (51.3 – 75.8) | 64.6 (60.2 – 72.7) | 68.4 (59.5 – 73.9) | 0.007 |
| Cardiac disease |  |  |  |  | <0.001 |
| ICM | 44 (26%) | 6 (46%) | 14 (34%) | 67 (48%) |  |
| DCM | 56 (33%) | 5 (38%) | 25 (61%) | 66 (47%) |  |
| Other | 70 (41%) | 2 (15%) | 2 (5%) | 7 (5%) |  |
| Primary prevention | 92 (54%) | 6 (46%) | 26 (65%) | 112 (82%) | <0.001 |
| Female gender | 42 (25%) | 3 (23%) | 6 (15%) | 18 (13%) | 0.055 |
| BMI (kg/m²) | 28.0 (24.6 – 31.6) | 29.5 (24.4 – 35.6) | 27.9 (24.9 – 33.0) | 26.7 (24.3 – 29.8) | 0.135 |
| LVEF (%) | 54.5 (45.0 – 55.0) | 45.0 (42.5 – 50.0) | 35.0 (31.8 – 40.0) | 28.0 (25.0 – 33.0) | <0.001 |
| CRT-D | 26 (15%) | 7 (54%) | 12 (30%) | 39 (28%) | 0.001 |
| NYHA |  |  |  |  | 0.012 |
| NYHA I | 59 (35%) | 6 (46%) | 8 (20%) | 26 (19%) |  |
| NYHA II | 71 (42%) | 2 (15%) | 19 (48%) | 64 (47%) |  |
| NYHA III | 39 (23%) | 5 (38%) | 13 (32%) | 47 (34%) |  |
| Atrial fibrillation |  |  |  |  | 0.496 |
| none | 112 (66%) | 6 (50%) | 26 (65%) | 76 (58%) |  |
| paroxysmal | 37 (22%) | 3 (25%) | 8 (20%) | 39 (30%) |  |
| permanent | 20 (12%) | 3 (25%) | 6 (15%) | 17 (13%) |  |
| NT-proBNP (ng/L) | 273.0 (112.0 – 702.5) | 1624.0 (682.0 – 2258.5) | 617.0 (276.0 – 1076.0) | 1228.0 (540.5 – 2129.0) | <0.001 |
| hsCRP (mg/L) | 2.0 (1.0 – 4.0) | 3.0 (1.5 – 5.2) | 3.0 (1.0 – 5.3) | 2.0 (1.0 – 4.0) | 0.325 |
| eGFR (mL/min) | 75.1 (60.0 – 93.3) | 72.5 (45.7 – 81.6) | 70.7 (54.6 – 81.4) | 64.0 (53.0 – 81.5) | 0.011 |
| Appropriate shock | 21 (12%) | 3 (23%) | 4 (10%) | 35 (26%) | 0.011 |
| Death | 16 (9%) | 4 (31%) | 8 (20%) | 38 (28%) | <0.001 |

Abbreviations:

BMI: body mass index; CRT-D: cardiac resynchronization therapy – defibrillator; DCM: dilated cardiomyopathy; eGFR: estimated glomerular filtration rate; hs-CRP: high sensitive C-reactive protein; ICM: ischemic cardiomyopathy; LVEF: left ventricular ejection fraction; NT-proBNP: brain natriuretic peptide; NYHA: New York Heart Association.

**Table S3.** Baseline characteristics by MTWA

|  | **Stable negative** | **Deterioration** | **Improvement** | **Stable non-negative** | **p-value** |
| --- | --- | --- | --- | --- | --- |
| n (%) | 85 (32%) | 51 (18%) | 53 (20%) | 79 (30%) |  |
| Age (y) | 60.3 (51.3 – 67.3) | 61.9 (55.0 – 72.8) | 63.5 (55.1 – 69.8) | 68.1 (59.4 – 74.4) | <0.001 |
| Cardiac disease |  |  |  |  | 0.007 |
| ICM | 28 (33%) | 17 (33%) | 18 (32%) | 31 (39%) |  |
| DCM | 21 (25%) | 19 (37%) | 27 (48%) | 35 (44%) |  |
| Other | 36 (42%) | 16 (31%) | 11 (20%) | 14 (18%) |  |
| Primary prevention | 45 (53%) | 30 (60%) | 37 (70%) | 56 (71%) | 0.073 |
| Female gender | 24 (28%) | 7 (14%) | 14 (26%) | 11 (14%) | 0.054 |
| BMI (kg/m²) | 27.1 (24.3 – 29.7) | 27.3 (24.9 – 30.2) | 29.0 (25.1 – 31.9) | 27.6 (25.2 – 31.6) | 0.312 |
| LVEF (%) | 53.3 (42.2 – 58.0) | 42.5 (33.3 – 55.0) | 37.0 (30.0 – 48.0) | 40.0 (30.0 – 49.5) | <0.001 |
| CRT-D | 9 (11%) | 11 (22%) | 12 (23%) | 19 (24%) | 0.096 |
| NYHA |  |  |  |  | 0.007 |
| NYHA I | 37 (43%) | 26 (51%) | 19 (36%) | 21 (27%) |  |
| NYHA II | 38 (45%) | 18 (35%) | 22 (41%) | 31 (39%) |  |
| NYHA III | 10 (12%) | 7 (14%) | 12 (23%) | 27 (34%) |  |
| Atrial fibrillation |  |  |  |  | 0.207 |
| none | 66 (79%) | 34 (68%) | 38 (76%) | 57 (73%) |  |
| paroxysmal | 18 (21%) | 16 (32%) | 10 (20%) | 21 (27%) |  |
| permanent | 0 (0%) | 0 (0%) | 2 (4%) | 0 (0%) |  |
| NT-proBNP (ng/L) | 166.0 (78.5 – 482.5) | 379.0 (123.0 – 710.0) | 437.5 (200.5 – 1503.3) | 653.5 (233.3 – 1444.0) | <0.001 |
| hsCRP (mg/L) | 2.0 (1.0 – 4.0) | 2.0 (1.0 – 4.0) | 2.0 (1.0 – 4.0) | 2.0 (1.0 – 4.0) | 0.799 |
| eGFR (mL/min) | 77.2 (67.4 – 96.5) | 72.9 (62.0 – 96.8) | 76.8 (57.2 – 87.4) | 64.8 (43.9 – 84.9) | 0.001 |
| Appropriate shock | 6 (7%) | 12 (24%) | 10 (19%) | 13 (16%) | 0.038 |
| Death | 8 (9%) | 6 (12%) | 5 (9%) | 15 (19%) | 0.278 |

Abbreviations:

BMI: body mass index; CRT-D: cardiac resynchronization therapy – defibrillator; DCM: dilated cardiomyopathy; eGFR: estimated glomerular filtration rate; hs-CRP: high sensitive C-reactive protein; ICM: ischemic cardiomyopathy; LVEF: left ventricular ejection fraction; MTWA: microvolt T-wave alternans; NT-proBNP: brain natriuretic peptide; NYHA: New York Heart Association.

**Table S4.** Baseline characteristics by PVC count

|  | **Stable ≤400/24h** | **Deterioration** | **Improvement** | **Stable >400/24h** | **p-value** |
| --- | --- | --- | --- | --- | --- |
| n (%) | 160 (41%) | 43 (11%) | 40 (10%) | 150 (38%) |  |
| Age (y) | 60.9 (53.7 – 70.5) | 66.7 (60.4 – 73.1) | 66.9 (59.4 – 72.9) | 67.9 (60.3 – 75.2) | <0.001 |
| Cardiac disease |  |  |  |  | <0.001 |
| ICM | 57 (35%) | 20 (47%) | 17 (42%) | 63 (42%) |  |
| DCM | 53 (32%) | 9 (21%) | 19 (48%) | 74 (49%) |  |
| Other | 54 (33%) | 14 (33%) | 4 (10%) | 14 (9%) |  |
| Primary prevention | 93 (58%) | 27 (63%) | 32 (80%) | 100 (67%) | 0.067 |
| Female gender | 44 (28%) | 8 (19%) | 6 (15%) | 21 (14%) | 0.024 |
| BMI (kg/m²) | 28.0 (24.5 – 31.1) | 27.6 (24.6 – 31.4) | 28.6 (24.7 – 31.7) | 26.9 (24.5 – 30.3) | 0.625 |
| LVEF (%) | 50.0 (35.0 – 55.0) | 44.8 (36.0 – 55.0) | 37.7 (27.8 – 45.0) | 36.0 (27.5 – 45.0) | <0.001 |
| CRT-D | 34 (21%) | 14 (33%) | 12 (30%) | 32 (21%) | 0.278 |
| NYHA |  |  |  |  | 0.003 |
| NYHA I | 61 (38%) | 12 (28%) | 8 (20%) | 31 (21%) |  |
| NYHA II | 65 (41%) | 22 (51%) | 14 (35%) | 75 (50% |  |
| NYHA III | 34 (21%) | 9 (21%) | 18 (45%) | 44 (29%) |  |
| Atrial fibrillation |  |  |  |  | 0.008 |
| none | 107 (67%) | 25 (61%) | 24 (60%) | 79 (54%) |  |
| paroxysmal | 43 (27%) | 11 (27%) | 10 (25%) | 36 (24%) |  |
| permanent | 9 (6%) | 5 (12%) | 6 (15%) | 32 (22%) |  |
| NT-proBNP (ng/L) | 427.5 (115.5 – 970.0) | 413.0 (120.0 – 956.0) | 515.5 (260.0 – 1621.8) | 854.0 (421.5 – 2074.5) | <0.001 |
| hsCRP (mg/L) | 3.0 (1.0 – 5.0) | 2.5 (1.0 – 5.3) | 3.0 (1.0 – 5.0) | 2.0 (1.0 – 4.0) | 0.117 |
| eGFR (mL/min) | 71.9 (57.3 – 88.7) | 67.4 (54.2 – 91.7) | 65.8 (55.0 – 78.0) | 68.3 (52.4 – 87.6) | 0.601 |
| Appropriate shock | 16 (10%) | 10 (23%) | 9 (22%) | 34 (23%) | 0.008 |
| Death | 18 (11%) | 7 (16%) | 7 (18%) | 42 (28%) | 0.002 |

Abbreviations:

BMI: body mass index; CRT-D: cardiac resynchronization therapy – defibrillator; DCM: dilated cardiomyopathy; eGFR: estimated glomerular filtration rate; hs-CRP: high sensitive C-reactive protein; ICM: ischemic cardiomyopathy; LVEF: left ventricular ejection fraction; NT-proBNP: brain natriuretic peptide; NYHA: New York Heart Association; PVC: premature ventricular complexes.

**Table S5.** Baseline characteristics by HRT

|  | **Stable normal** | **Deterioration** | **Improvement** | **Stable abnormal** | **p-value** |
| --- | --- | --- | --- | --- | --- |
| n (%) | 59 (30%) | 26 (13%) | 33 (16%) | 82 (41%) |  |
| Age (y) | 57.9 (44.5 – 66.6) | 63.9 (56.8 – 70.9) | 63.2 (53.8 – 69.2) | 67.1 (60.8 – 72.8) | <0.001 |
| Cardiac disease |  |  |  |  | 0.001 |
| ICM | 20 (34%) | 11 (42%) | 13 (37%) | 39 (47%) |  |
| DCM | 16 (27%) | 12 (46%) | 14 (40%) | 37 (45%) |  |
| Other | 23 (39%) | 3 (12%) | 8 (23%) | 7 (8%) |  |
| Primary prevention | 25 (42%) | 15 (58%) | 21 (64%) | 63 (78%) | <0.001 |
| Female gender | 15 (25%) | 4 (15%) | 13 (39%) | 13 (16%) | 0.044 |
| BMI (kg/m²) | 25.4 (22.4 – 58.6) | 28.5 (26.2 – 31.5) | 29.0 (25.4 – 31.2) | 28.2 (25.9 – 31.4) | 0.005 |
| LVEF (%) | 50.0 (41.0 – 58.0) | 43.3 (35.0 – 51.9) | 37.5 (30.0 – 53.0) | 36.0 (28.0 – 47.3) | <0.001 |
| CRT-D | 1 (2%) | 4 (15%) | 4 (12%) | 19 (23%) | 0.001 |
| NYHA |  |  |  |  | <0.001 |
| NYHA I | 35 (59%) | 6 (23%) | 13 (39%) | 16 (20%) |  |
| NYHA II | 18 (31%) | 12 (46%) | 14 (42%) | 42 (51%) |  |
| NYHA III | 6 (10%) | 8 (31%) | 6 (19%) | 24 (29%) |  |
| Atrial fibrillation |  |  |  |  | 0.110 |
| none | 52 (88%) | 16 (62%) | 26 (79%) | 59 (76%) |  |
| paroxysmal | 7 (12%) | 9 (35%) | 6 (18%) | 17 (22%) |  |
| permanent | 0 (0%) | 1 (4%) | 1 (3%) | 2 (3%) |  |
| NT-proBNP (ng/L) | 202.5 (102.0 – 623.3) | 380.0 (145.3 – 1227.8) | 477.0 (284.5 – 1142.0) | 502.5 (196.0 – 1291.8) | 0.028 |
| hsCRP (mg/L) | 2.0 (1.0 – 3.0) | 2.0 (2.0 – 6.0) | 2.2 (1.0 – 3.8) | 3.0 (1.0 – 5.2) | 0.277 |
| eGFR (mL/min) | 84.4 (70.6 – 102.6) | 65.8 (46.8 – 79.6) | 71.5 (61.8 – 88.5) | 70.8 (53.7 – 89.3) | <0.001 |
| Appropriate shock | 11 (19%) | 4 (15%) | 5 (15%) | 15 (18%) | 0.978 |
| Death | 1 (2%) | 3 (12%) | 2 (6%) | 24 (29%) | <0.001 |

Abbreviations:

BMI: body mass index; CRT-D: cardiac resynchronization therapy – defibrillator; DCM: dilated cardiomyopathy; eGFR: estimated glomerular filtration rate; HRT: heart rate turbulence; hs-CRP: high sensitive C-reactive protein; ICM: ischemic cardiomyopathy; LVEF: left ventricular ejection fraction; NT-proBNP: brain natriuretic peptide; NYHA: New York Heart Association.
